# Whole Cottonseed as an Effective Strategy to Mitigate Enteric Methane Emissions in Cattle Fed Low-Quality Forages †

**DOI:** 10.3390/ani15060819

**Published:** 2025-03-13

**Authors:** Olegario Hernández, Agustín López, Maria Esperanza Ceron-Cucchi, Cham Donald AdégbéÏga Alabi, Cecilia Loza, Ana Veronica Juárez Sequeira, Héctor Miguel Fissolo, Elisa Mariana García, José Ignacio Gere

**Affiliations:** 1 Estación Experimental Agropecuaria Santiago del Estero, Instituto Nacional de Tecnología Agropecuaria (INTA), Jujuy 850, Santiago del Estero 4200, Argentina; lopez.agustin@inta.gob.ar (A.L.); fissolo.hectormiguel@inta.gob.ar (H.M.F.); 2 Facultad de Agronomía y Agroindustrias, Universidad Nacional de Santiago del Estero, Av. Belgrano (S) 1912, Santiago del Estero 4200, Argentina; anajuarezsequeira@gmail.com; 3 Consejo Nacional de Investigaciones Científicas y Técnicas (CONICET), Ciudad Autónoma Buenos Aires C1033AAJ, Argentina; ceroncucchi.maria@inta.gob.ar (M.E.C.-C.); marian_sgo@yahoo.com.ar (E.M.G.); 4 Instituto de Patobiología Veterinaria (IPVet), Instituto Nacional de Tecnología Agropecuaria (INTA-CONICET), Hurlingham C1417AZE, Argentina; 5 Unidad de Investigación y Desarrollo de las Ingenierías, Universidad Tecnológica Nacional, Facultad Regional Buenos Aires (UTN FRBA), Ciudad Autónoma Buenos Aires C1179AAQ, Argentina; cloza@fagro.edu.uy; 6 Laboratory of Ecology, Health and Animal Production (LESPA), Faculty of Agronomy (FA), University of Parakou (UP), Parakou O1 BP 123, Benin; donach2@gmail.com; 7 Departamento de Producción Animal y Pasturas, Facultad de Agronomía, Universidad de la República, Montevideo 12900, Uruguay; 8 Instituto de Ciencias Químicas (ICQ), Facultad de Agronomía y Agroindustrias (FAyA), Universidad Nacional de Santiago del Estero (UNSE), Santiago del Estero 4200, Argentina

**Keywords:** livestock production, beef cattle, tropical forage, SF6 tracer technique, by-products

## Abstract

This study evaluated enteric methane (CH₄) emissions, dry matter intake (DMI), and performance in rearing beef heifers fed either a Guinea-grass-only diet (0WCS) or Guinea grass supplemented with whole cottonseed (WCS) at 0.5% of body weight (BW). Twenty-four Braford heifers were randomly allocated into four pens (three animals per pen) per treatment over two experimental periods. Methane emissions were measured using the SF₆ tracer technique. Heifers receiving WCS supplementation produced 29% less CH₄ (120.64 vs. 169.54 g/day for 0.5WCS and 0WCS, respectively; *p* = 0.02) and showed a 22% reduction in CH₄ yield (7.30% vs. 9.41% of gross energy intake; *p* = 0.02). Methane intensity was 33% lower in supplemented heifers (0.37 vs. 0.55 g CH₄/kg BW; *p* = 0.01). However, WCS supplementation significantly reduced total DMI and forage DMI (*p* = 0.01 and *p* < 0.01, respectively). In terms of performance, heifers in the 0.5WCS group gained 0.28 kg/day, while those in the 0WCS group lost 0.10 kg/day. These results indicate that WCS supplementation mitigates CH₄ emissions while improving weight gain in beef heifers fed low-quality forage diets, making it a promising strategy for enhancing the sustainability of beef cattle production systems.

## Simple Summary

This study evaluates the effects of whole cottonseed (WCS) supplementation on methane (CH₄) emissions, dry matter intake (DMI), and performance in beef heifers fed low-quality forage diets. WCS was supplemented at 0.5% of body weight (BW). Results demonstrated that WCS supplementation reduced CH₄ emissions by 29% (g/day) and CH₄ yield by 22% (percentage of gross energy intake) compared to a forage-only diet. Methane intensity (g CH₄/kg BW) decreased by 33%. However, DMI was significantly reduced, indicating a substitution effect accompanied by a depression intake. These findings support the potential of WCS to mitigate enteric CH₄ emissions in beef cattle systems reliant on low-quality forages.

## 1. Introduction

Human activities account for approximately 60% of global methane (CH₄) emissions, with enteric CH₄ production and manure management contributing about 32% of anthropogenic emissions [1]. When combined with non-anthropogenic sources, these emissions account for 19% of total global CH₄ emissions [2]. Methane is a potent greenhouse gas (GHG) with a global warming potential (GWP) 21 to 26 times greater than carbon dioxide (CO₂), making it a significant driver of climate change. Additionally, CH₄ production in the rumen represents an energy loss for ruminants, further exacerbating its environmental and economic implications [3,4].

Numerous studies [5,6] have established a positive correlation between CH₄ production and the intake of digestible cellulose, hemicellulose, and non-fiber carbohydrates, whereas fat intake shows a negative correlation. Lipid supplementation has emerged as a promising strategy for mitigating enteric CH₄ emissions across various ruminant species [7,8,9]. This reduction is primarily achieved through three mechanisms: (i) lipids act as an alternative hydrogen sink as unsaturated fatty acids undergo biohydrogenation in the rumen [10]; (ii) supplemental lipids often reduce dry matter intake (DMI), indirectly lowering CH₄ emissions [11,12,13]; and (iii) specific lipids, particularly medium-chain fatty acids, can alter the rumen microbiome by suppressing protozoa and archaea populations [14,15].

Industrial by-products such as whole cottonseed (WCS) are rich in fat (~18–19% EE), especially polyunsaturated fatty acids (PUFAs). In this sense, from the total lipids in WCS, 77.68% are PUFA [16]. These by-products are widely used as supplements to enhance the average daily gain (ADG) of beef cattle grazing on low-quality forages [17], primarily due to their high concentrations of rumen-degradable protein (RDP) and metabolizable energy (ME) [18,19]. The high fat content of these feedstuffs also suggests their potential as mitigators of enteric CH₄ emissions. However, their effects on CH₄ emissions in forage-based diets, particularly under tropical and subtropical conditions, remain poorly understood [7,10].

In northwestern Argentina, introduced tropical pastures are widely used for livestock production, with Guinea grass (*Megathyrsus maximus* cv. Gatton panic) being the most prominent [20]. This species produces over 60% of its annual forage yield during the summer rainy season [21]. During winter, low-quality deferred forage is commonly utilized [22], leading to nutritional limitations for livestock. Under these conditions, low forage intake and digestibility are the primary constraints on animal productivity [23], as both energy and protein supply become severely restricted [24]. To address these challenges, winter protein or protein-energy supplementation has been widely implemented as a management strategy to prevent weight loss and even improve ADG in cattle consuming low-quality feedstuffs [25,26].

Argentina’s provinces of Santiago del Estero and Chaco account for 79% of the country’s cotton production. Whole cottonseed (WCS), a by-product of the textile industry, is commonly incorporated into ruminant diets for beef cattle [27]. Additionally, its low cost and proximity to farms make WCS an attractive feed option for both small- and large-scale farmers. Given its nutritional composition, WCS has the potential to reduce enteric CH₄ emissions while enhancing animal performance, particularly in diets based on low-quality forages.

This study aimed to assess the effects of WCS supplementation on intake, digestion, animal performance, and enteric CH₄ emissions in Braford crossbred heifers consuming low-quality forage diets. The preliminary hypothesis was that WCS supplementation would reduce enteric CH₄ production while enhancing animal performance under these conditions.

## 2. Materials and Methods

The experiment was conducted over 85 days (from 30 June 2022, to 23 September 2022) during the dry, cold season at the INTA Santiago del Estero Research Station (28°01′32″ S, 64°13′58″ W; 170 m elevation). All experimental procedures were approved by the INTA Tucumán-Santiago Institutional Animal Care and Use Committee (Approval No. 03/22). Average temperature and relative humidity during the period in which this study was carried out were 14.92 °C and 56.20%, respectively, with no rainfall.

### 2.1. Animals, Experimental Design and Diets

Twenty-four crossbred Braford beef heifers (318.21 ± 31.18 kg BW) were randomly assigned to four pens per treatment (three heifers per pen), across two measurement periods, in a completely randomized design, with each pen considered the experimental unit. Each pen measured 240 m² (12 × 20 m).

Two dietary treatments were evaluated: 0WCS—Guinea grass hay (GGH; *Megathyrsus maximus* cv. Gatton panic) with no supplementation (forage-to-concentrate ratio: 100:0); and 0.5WCS—Guinea grass hay supplemented with whole cottonseed (WCS) at 0.5% of BW (as-fed basis), resulting in a forage-to-concentrate ratio of 74:26. Animals were fed once daily at 7:00 a.m. Guinea grass hay was offered in a 6 m long canvas feed bunk, while WCS was provided in individual 1.5 m plastic feed bunks. Water was available *ad libitum*.

Feed intake was determined by weighing feed refusals separately for hay and supplement. The WCS used was untreated, consisting of small seeds surrounded by lint. The chemical composition of GGH and WCS is presented in Table 1.

The experiment was divided into two measurement periods. Period 1 (18 to 40 d) and Period 2 (59 to 81 d) both consisted of 14 d for treatment adaptation, 5 d for feed intake measurement, 4 d for enteric CH_4_ emission monitoring, and 3 d for digestion evaluation. Animals consumed the treatments uninterruptedly during the whole experiment (1–85 d).

### 2.2. Feed Intake and Digestibility

Feed intake was calculated as the difference between feed offered (kg DM) and feed refusals (kg DM). Nutrient intake was corrected for nutrient concentration in both offered feeds and refusals.

Total tract digestibility was estimated using acid detergent insoluble ash as an internal marker, following Cochran and Galyean’s method [28]. Representative fecal grab samples (around 300–400 g per sample) were collected directly from the rectum of the animals to ensure accuracy in the sample collection process. Fecal grab samples were collected every 6 h during the final 3 days of each evaluation period (days 38 to 40 for Period 1 and days 79 to 81 for Period 2). Sampling times were rotated by 3 h daily to cover a 24 h cycle and minimize diurnal variation in marker excretion. For operative and equipment problems, we had to measure only in Period 2. Nevertheless, due to the stability in chemical composition of hay and WCS, we decided to use it as a global parameter.

Animals were weighed at the beginning and end of the experiment, and average daily gain (ADG) was estimated as the difference between final and initial body weight, divided by the duration of the experiment (i.e., 85 days). Weights were recorded without a fasting period, and animals had unlimited access to water throughout the experiment.

### 2.3. Enteric Methane Emission

Enteric CH₄ emission (g CH₄/day) was measured using the sulfur hexafluoride (SF₆) tracer technique, as proposed by Johnson et al. [29] and adapted for extended periods [30,31]. Samples were collected over 4 days, on days 33 to 37 for Period 1 and days 74 to 78 for Period 2.

Permeation tubes containing 1.83 ± 0.17 g of SF₆ were dosed orally using a custom dispenser. Tubes were pre-weighed weekly for 8 weeks while stored at 39 °C to estimate permeation rates (average: 11.18 ± 2.14 mg/d).

**Figure 1:**
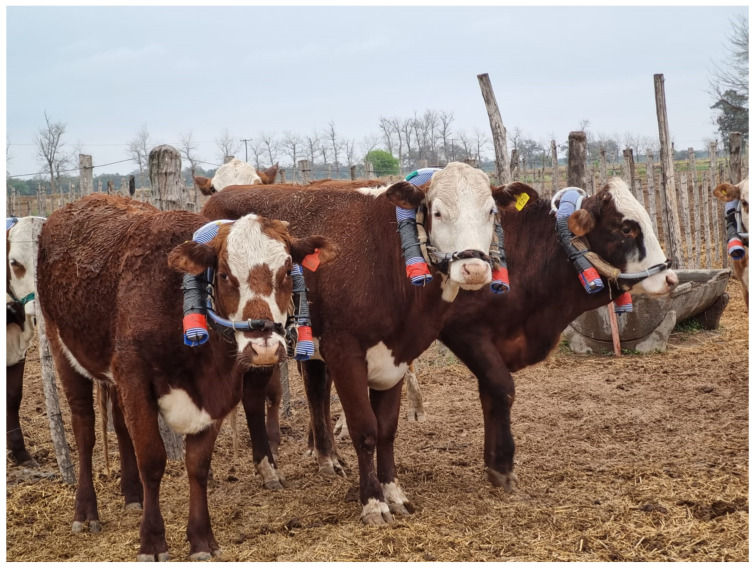
Experimental animals equipped with devices for measuring enteric CH_4_ emissions using the SF_6_ tracer technique. Each animal is fitted with two sample collection systems, housed within a blue corrugated tube designed to contain the equipment. The tube is securely attached to the muzzle.

The sample collection system included two 0.5 L stainless steel containers, while the flow regulator consisted of a 10 cm metal capillary, with a 5 mm segment compressed to achieve a target flow rate of 0.05 mL/min (the restrictor was calibrated to maintain a pressure between 0.4 and 0.6 bars at the end of the sampling period). Air samples were collected continuously over 4 days using 0.5 L stainless-steel containers placed near the nostrils of each animal (Figure 1). Before sampling, the containers were cleaned with nitrogen gas (99.9% purity) and evacuated to a pressure of −0.99 bar relative to atmospheric pressure. The restrictor and the sampling line were housed in 5 cm polyethylene tubing (12 mm inner diameter), with a polyester fabric cover to prevent blockage from water or dust. Background air samples were collected near the pens for CH₄ and SF₆ concentration corrections.

The CH_4_ and SF_6_ concentrations were analyzed via gas chromatography (Perkin Elmer 600, Kansas City, MO, USA) at the Pathobiology Veterinary Institute (CICVyA, INTA, Argentina) according to the methodology described by Gere et al. [32]. Methane production was calculated using the following equation:

$${CH}_{4}\left(g/d\right)=PR{SF}_{6}\left(g/d\right)\times \left(\frac{\left[{CH}_{4}-{CH}_{4B}\right]}{\left[{SF}_{6}-{SF}_{6B}\right]}\right)\times \frac{MWCH₄}{MWSF₆}$$

where: CH₄ emission rate (g/d); CH₄, SF₆—gas concentrations from exhaled air; CH_4B_, SF_6B_—background gas concentrations; PRSF₆—SF₆ permeation rate (g/d); MWCH₄, MWSF₆—Molecular weights of CH₄ and SF₆.

### 2.4. Statistical Analyses

The experimental design was completely randomized. Data were analyzed using the software INFOSTAT 2020 (Di Rienzo et al., 2020) [33] with an interface with R through mixed linear models. Each pen was considered an experimental unit. WCS supplementation levels were considered fixed effects in each period; the pen was a random effect. Multiple comparisons between means were performed using the LSD Fisher test (*p* < 0.05). The following model was fitted to the data set for all variables:

Yij = µ + T_i_ + P_j_ + A_k_ + (T × P)_ij_ + ε_ijk_

where Y_ij_ is the response to treatment, µ is the overall mean, Ti is the fixed effect of treatment i, Pj is the fixed effect of period, Ak is the random effect of Pen k, (T × P)ij is the interaction between treatment and period, and ɛ_ijk_ is the experimental error.

## 3. Results and Discussion

### 3.1. Feed Intake

As shown in Table 2, total dry matter intake (TDMI) decreased by 15.40% in Period 1 and 3.70% in Period 2 with WCS supplementation (*p* < 0.01). This reduction indicates a substitution effect, with a depression in DMI. A similar decline was observed in forage dry matter intake (FDMI), which decreased 36.85% and 30.30% in Periods 1 and 2, respectively (*p* < 0.01). No effect was observed in gross energy intake (GEI) in both periods (*p* = 0.47). TDMI and FDMI expressed as % of BW showed significant decrease with WCS supplementation (*p* < 0.001). It is important to note that 0.5WCS heifers actually consumed 0.38 and 0.42% of body weight for Period 1 and 2, respectively, which is less than the targeted level of 0.5% of BW. Additionally, total organic matter intake (TOMI), neutral detergent fiber intake (NDFI), and acid detergent fiber intake (ADFI) were also lower in supplemented animals (*p* < 0.01).

In contrast, crude protein intake (CPI) and ether extract intake (EEI) increased significantly (*p* < 0.01) with supplementation, as expected due to the higher protein and fat content of WCS. The period effect (*p* < 0.05) could be explained by variations in animal weight across periods. The treatment–period interaction did not reach statistical significance (*p* > 0.05).

Previous research suggests that WCS supplementation should be around 0.5% of body weight (BW), corresponding to approximately 2.3 to 3.2 kg WCS per cow per day [34,35]. In this study, WCS supplementation averaged 23% of DM and reduced total DM intake (TDMI), forage DM intake (FDMI), and total organic matter intake (TOMI), consistent with the substitution effect caused by the high lipid content of WCS (18.71% ether extract). Total DMI reduction was explained by reductions in WCS intake and forage intake. This observation aligns with the well-established finding that lipid supplementation generally reduces DMI across various types of diets [11,12,13]. Specifically, it has been widely reported that, in growing cattle, the supplementation with WCS above 0.33% BW (≅ 15% DM) causes a rapid drop in voluntary feed intake [34,35].

Studies in beef cows fed Bermuda grass hay [35] and in Zebu cattle fed rice-straw-based diets [36] reported reductions in DMI only at higher levels of WCS supplementation (1% BW) than those used in this study. Conversely, Beck et al. [9] observed a decrease in FDMI but an increase in TDMI with WCS supplementation at 0.5% BW, reflecting a balance between substitution and addition effects. Given this, Bradford et al. [37] explain that diets with more than 6% of rumen-degradable fat (especially unsaturated forms) inhibit fiber digestion by ruminal microbes’ activity, and in some cases, decrease feed intake. Similarly, Panahiha et al. [38] suggest that increasing the intake of unsaturated fatty acids (UFA) can lead to a decrease in DMI and organic matter digestibility in the rumen. Harvatine and Allen [39] indicate that the supplementation of UFAs may reduce intake by producing fatty acids that serve as physiological signals, prompting a decrease in meal size or an increase in the intervals between meals. Additionally, Kargar et al. [40] found that higher proportions of UFAs in supplemental fat enhance the hypophagia effects of fat. An earlier study by Drackley et al. [41] observed that postruminal infusion of UFAs implicated additional postruminal mechanisms in feed intake regulation. Further evidence from multiple studies [42,43,44] suggests that UFA supply enhances secretion of gut peptides such as glucagon-like peptide 1 and cholecystokinin, both of which contribute to feed intake suppression.

Nevertheless, other studies [15,45,46] found no differences in DMI or forage intake, likely due to variations in experimental conditions.

The higher EE content of 0.5WCS diets vs. 0WCS diets compensated for the negative effect of lipid supplementation on DMI, resulting in a similar GEI between 0.5WCS heifers and those fed 0WCS.

Despite the reduced intake of dry matter and forage, CPI and EEI were higher in the supplemented group, reflecting the increased availability of protein and fat in WCS. This finding aligns with Beck et al. [9], who reported similar increases in protein and energy intake with WCS supplementation. Research on forage intake and supplementation in cattle has shown that energy supplementation can lead to a substitution effect, where forage intake decreases as supplement intake increases [47,48] This effect is more evident with higher-quality forages and increased levels of supplementation [48,49]. However, the substitution rate can vary depending on factors such as forage quality, supplement type, and level of supplementation [47,48]. Particularly, energy supplementation can improve dry matter digestibility. Nevertheless, it may not always enhance body weight gain in cattle on tropical pastures during the rainy season, so some strategies to minimize substitution effects may include balancing protein and energy levels in supplements [48] and considering the metabolizable energy intake from supplements [50]. Additionally, high-lipid supplements can affect substitution rates, with rumen-degradable protein availability and lipid intake playing crucial roles [50].

### 3.2. Digestion

As shown in Table 3, WCS supplementation improved crude protein (CP) digestibility (CPD) and ether extract digestibility (EED) (*p* < 0.01). However, no significant differences were observed in dry matter digestibility (DMD), neutral detergent fiber digestibility (NDFD), or acid detergent fiber digestibility (ADFD).

A numerical difference of 9% in total tract digestion was noted; however, due to the high variation, it was not statistically different. This lack of effect on DMD is consistent with previous studies on dairy cows fed total mixed rations (TMR) [51] and beef cattle on high-forage diets [35]. In contrast, Chuntrakort et al. [36] observed a reduction in DMD with higher WCS supplementation levels (1% BW). Similarly, Hill et al. [35] reported negative effects on digestibility when WCS was offered as a free choice. In addition, in this study, the total fat content of the diet containing WCS (5.7 and 4.8% DM for Period 1 and 2, respectively) was below the 6–7% concentration expected to cause depressions in DM digestibility [52,53]. In this sense, Ismartoyo [54] suggests that the inclusion of WCS in ruminant diets reduces dry matter and fiber digestibility due to a negative effect on the numbers and activity of rumen microbes. In addition, this author suggests that it is not clear whether fat content or gossypol and/or a combination of both decrease the dry matter and fiber digestibility. It has been reported that the presence of gossypol might have contributed to the reduction of the number of rumen microbes and the degradation of grass hay. However, this effect was not observed in our experiment. Other studies [55] reported that there are factors that limit the use of WCS in a ration, and one of these factors is the high concentration of fat, which can lead to negative effects on fiber digestion due to the negative effects of the free oil on the microbial population in the rumen.

The increased CP digestibility in the 0.5WCS group aligns with some studies [53,56], although others found no significant differences between the control and supplemented groups [36,51,57]. The unaffected NDFD is consistent with findings by Hill et al. [35], Nogueira [51], and Beck et al. [9], although Chuntrakort et al. [36] reported reductions with WCS supplementation. Similarly, ADFD showed no differences between treatments, corroborating earlier reports [36,51], although Beck et al. [9] observed a decline at 0.5% BW supplementation. We could attribute the increase in crude protein digestibility in our experiment to the higher crude protein in the diet.

The doubling of EED in the 0.5WCS group, compared to controls, is consistent with Chuntrakort et al. [36]. Nogueira et al. [51] also reported a 17% increase in EED with WCS supplementation in dairy cows fed sugarcane bagasse. Previous literature regarding WCS supplementation [56] evaluated three levels of supplementation (0, 15, and 30%) and reported an increase in EE digestibility as supplementation increased in diet, suggesting that the increased digestibility of ether extract with WCS can be attributed to the dilution of the metabolic fecal fat with a dietary fat of high true digestibility.

### 3.3. Animal Performance

WCS supplementation significantly improved ADG (*p* < 0.01), with supplemented animals gaining 340 g/day more than controls (Table 3). This resulted in a final body weight up to 44 kg higher in the supplemented group. Control animals lost 100 g/day, while 0.5WCS animals gained 280 g/d.

The control weight loss contrasts with findings from other studies [9,35,46], where non-supplemented groups showed weight gain, albeit lower than supplemented groups. This discrepancy likely reflects differences in baseline diet quality. In our study, based on the guidelines established by NASEM [58], the 0WCS treatment failed to meet the animals’ maintenance requirements (CP: 416 g/d and NEm: 5.49 Mcal/d). These animals would need to increase their voluntary intake by around 40% to avoid weight loss, which is practically impossible due to the physical limitations of the rumen, given the quality of the forage provided. However, the 0.5%WCS treatment increased the CP intake by 54% compared to the control (535.6 vs. 348.1 g/d, respectively), providing 1.18 Mcal NEg available for weight gain. According to NASEM [58], the observed ADG was slightly lower than the predicted ones (0.280 vs. 0.306 kg/d, respectively).

The observed increase in ADG underscores the dual benefits of WCS as a source of energy and metabolizable protein. These results highlight the need for optimizing supplementation strategies, particularly in low-quality forage-based systems, to improve animal performance.

### 3.4. Enteric Methane Emission

As shown in Table 4, enteric CH₄ emissions (g/d) decreased by approximately 30% in both periods with WCS supplementation. Methane yield (g CH₄/kg DMI) showed a tendency to decrease (*p* < 0.01). Methane intensity (g CH₄/kg BW) significantly declined by 33% and 36% in Period 1 and 2, respectively, while Ym (%) decreased by 22% and 30% (*p* < 0.05). The period effect observed in enteric CH₄ emissions (g/d) and CH₄ intensity (g CH₄/kg BW and g/kg BW⁰·⁷⁵) (*p* < 0.05) could be attributed to variations in animal weight across periods. A possible explanation for the variation in CH₄ emissions between periods is the influence of different feed intake levels, as higher DMI results in greater CH₄ emissions, due to the increased availability of fermentable substrate. This relationship was demonstrated by Boadi and Wittenberg [59], who found that the amount of feed provided directly affected CH₄ production, with DMI showing a strong correlation with CH₄ emissions. Indeed, when CH₄ emissions are expressed per kg of DMI, the difference between periods disappears (*p* > 0.10), suggesting that the observed variation is primarily driven by differences in feed intake rather than changes in CH_4_ yield per unit of intake. There were no statistically significant interactions found between treatment and period (*p* > 0.10).

These reductions in CH_4_ emissions in the 0.5 WCS treatment are consistent with findings by Beck et al. [9] and Chuntrakort et al. [36], who reported similar long-term effects. Specifically, Beck et al. [15] observed the lowest CH₄ emissions with 0.6% WCS supplementation. Other studies [7,45,60], also noted reductions in CH₄ emissions with WCS inclusion, particularly when it replaced a substantial portion of the diet. Mechanisms for reduced CH₄ emissions include disruption of methanogens, enhanced biohydrogenation of unsaturated fatty acids, and a shift in rumen fermentation towards propionate production [61,62,63]. Additionally, high-fat diets reduce substrate availability for methanogenesis, as noted by Knapp et al. [64] and Muñoz et al. [65]. In a meta-analysis, Eugene et al. [11] described that several factors could explain the depressing effect of lipid supplementation on CH_4_ production. These include the chain length and the degree of unsaturation of fatty acids of the lipid supplement. Moreover, it was shown through *in vitro* experiments that medium-chain fatty acids (8 to 16 C), such as lauric (C12:0) and myristic (C14:0) acids, caused a greater decrease in CH_4_ production compared with short (≤8 C) or long (≥18 C) fatty acids [66]. Others suggested that depression in CH_4_ production due to lipid supplementation includes a reduction in the amount of organic matter fermented in the rumen [67] and the inhibitory effect of lipids on ruminal microorganisms’ activity [68].

The assessment of the feasibility and viability of using additives to mitigate enteric CH_4_ emissions requires determination of their ability to produce a long-term effect [69]. An interesting finding of this study is that the mitigation effect was sustained throughout the 27 days between the two enteric CH₄ emission monitoring periods, with a reduction of approximately 30%.

Studies conducted in Argentina on pasture-fed beef cattle have reported variable Ym values, ranging from 4.0% to 8.2% [70] and from 4.3% to 6.2% [71]. The CH₄ yield observed in this study, particularly in the control treatment, exceeded the average values reported in these previous works as well as the IPCC default value of 6.5%. This underscores the urgent need for targeted mitigation strategies in cattle systems relying on low-quality forages, such as Guinea grass.

These findings demonstrate the potential of WCS as an effective CH₄ mitigation strategy in ruminant diets. Future research should also assess the effects of WCS supplementation on animal performance, rumen fermentation, microbiology, and the scalability of this approach in commercial livestock systems.

## 4. Conclusions

Due to the chemical composition (characterized by lower fermentable dry matter and high lipid content) of whole cottonseed and the lower total feed intake in the WCS group, heifers that received WCS showed a methane mitigation effect. Compared to the un-supplemented group, WCS supplementation decreased total feed intake by 19%, but the total energy intake was higher in the WCS group, resulting in improvements in average daily gain. Furthermore, WCS supplementation may contribute to reducing enteric methane emissions and aligning livestock production systems with sustainability goals by addressing both productivity and environmental challenges.

This study aligns livestock production systems with sustainability goals by addressing both productivity and environmental challenges. Future research should explore the scalability of WCS supplementation across diverse production environments and evaluate its long-term effects on animal health, rumen microbiota, and overall system sustainability.

## Figures and Tables

**Table 1:** Chemical composition of Guinea grass hay (GGH) and whole cottonseed (WCS).

Item	Ingredients	
	Guinea Grass Hay	Whole Cottonseed
DM, %	84.60	77.70
OM, %	90.30	93.32
CP, % DM	5.29	20.97
NDF, % DM	68.48	50.68
ADF, % DM	51.86	44.14
EE, % DM	1.27	18.71
Ash, % DM	9.70	6.68

DM: dry matter, CP: crude protein, NDF: neutral detergent fiber, ADF: acid detergent fiber, EE: ether extract, OM: organic matter, ash: remaining matter after incineration at 600 °C.

**Table 2:** Effect of whole cottonseed supplementation on heifers fed low-quality forage on intake and average daily gain.

	Period 1		Period 2			*p*-Value ^3^		
	Treatments ^1^		Treatments		SEM ^2^	T	P	T × P
	0WCS	0.5WCS	0WCS	0.5WCS				
Body weight (kg) *	310.84	324.33	304.25	332.50	13.56	0.14	0.0095	0.0001
DM intake (kg/d)								
Forage	5.78	3.65	6.58	5.05	0.12	<0.001	<0.001	0.02
WCS	0	1.25	0	1.28	0.03	<0.001	0.42	0.42
Total	5.78	4.89	6.58	6.34	0.12	<0.01	<0.001	0.02
GEI (MJ/d)	99.12	91.51	112.80	116.55	2.19	0.47	<0.001	0.02
DM intake (% BW)								
Forage	1.87	1.10	2.15	1.65	0.05	<0.001	<0.001	0.03
WCS	0	0.38	0	0.42	4.7×10^-3^	<0.001	0.002	0.002
Total	1.87	1.48	2.15	2.07	0.05	<0.001	<0.001	0.02
Nutrient intake (g/kg BW^0.75^)								
DM	78.23	62.99	90.44	79.00	2.41	<0.001	0.001	0.29
OM	70.64	57.35	81.66	71.81	2.11	<0.001	0.001	0.29
CP	4.55	6.46	5.27	7.37	0.18	<0.01	<0.01	0.51
NDF	59.14	44.49	68.36	56.55	1.79	<0.01	<0.01	0.27
ADF	38.71	29.90	44.73	37.85	1.18	<0.01	<0.01	0.24
EE	1.00	3.58	1.16	3.81	0.08	<0.01	0.05	0.68

^1^ 0WCS: Guinea grass hay with no supplementation. 0.5WCS: Guinea grass hay with whole cottonseed offered at 0.5% of the body weight. ^2^ SEM: standard error of the mean. ^3^ T: treatment, P: period, T × P: interaction between T and P. * Initial body weight per each treatment. WCS: whole cottonseed, GEI: gross energy intake, BW^0.75^: metabolic body weight, DM: dry matter, OM: organic matter, CP: crude protein, NDF; neutral detergent fiber, ADF: acid detergent fiber, EE: ether extract.

**Table 3:** Effect of whole cottonseed supplementation on animal performance and digestibility of heifers fed low-quality forage.

	Treatments ^1^		SEM ^2^	*p*-Value
	0WCS	0.5WCS		
Initial BW, kg *	310.84	324.33	9.02	0.35
Final BW, kg **	304.25	347.83	9.97	<0.01
ADG, kg/d	−0.10	0.28	0.02	<0.01
DMD, %	43.07	47.08	4.24	0.47
CPD, %	35.55	65.03	4.60	<0.01
NDFD, %	46.80	46.53	4.33	0.97
ADFD, %	42.94	42.26	4.69	0.92
EED, %	43.28	86.05	6.89	<0.01

^1^ 0WCS: Guinea grass hay with no supplementation. 0.5WCS: Guinea grass hay with whole cottonseed offered at 0.5% of the body weight. ^2^ SEM: standard error of the mean. * Initial body weight of the experiment, ** Final body weight of the experiment, BW: body weight, ADG: average daily gain, DMD: dry matter digestibility, CPD: crude protein digestibility, NDFD: neutral detergent fiber digestibility, ADFD: acid detergent fiber intake, EED: extract digestibility. Animals received the diets uninterruptedly throughout the experiment (1–85 d).

**Table 4:** Effect of whole cottonseed supplementation on heifers fed low-quality forage on methane production.

	Period 1		Period 2			*p*-Value ^3^		
	Treatments ^1^		Treatments		SEM ^2^	T	P	T × P
	0WCS	0.5WCS	0WCS	0.5WCS				
CH_4_ (g/d)	169.56	120.64	209.02	151.11	13.89	<0.01	0.04	0.76
CH_4_ (g/kgDMI)	29.23	24.72	27.04	19.83	1.89	0.02	0.11	0.50
CH_4_ (g/kg BW)	0.55	0.37	0.69	0.44	0.04	<0.01	0.05	0.45
CH_4_ (g/kg BW^0.75^)	2.30	1.55	2.87	1.88	0.17	<0.01	0.04	0.52
Ym	9.41	7.30	10.24	7.16	0.74	<0.01	0.97	0.74

^1^ 0WCS: Guinea grass hay with no supplementation. 0.5WCS: Guinea grass hay with whole cottonseed offered at 0.5% of the body weight. ^2^ SEM: standard error of the mean. ^3^ T: treatment, P: period, T × P: interaction between T and P. CH_4_: methane, DMI: dry matter intake, BW: body weight. BW^0.75^: metabolic body weight, Ym: methane yield as a percentage of gross energy intake.

## Data Availability

The original contributions presented in this study are included in the article. Further inquiries can be directed to the corresponding author.
